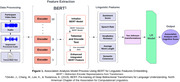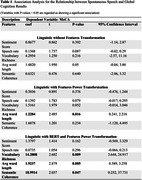# Transformer‐based Deep Learning Architecture Improves Detection of Associations between Spontaneous Speech Language Markers and Cognition

**DOI:** 10.1002/alz.089917

**Published:** 2025-01-09

**Authors:** Pooyan Mobtahej, Sam Gouron, Rojan Javaheri, Annelisse El‐Khoury, Anne‐Marie C Leiby, David L Sultzer, S. Ahmad Sajjadi

**Affiliations:** ^1^ University of California, Irvine, Irvine, CA USA; ^2^ University of California, Irvine, CA USA; ^3^ The UC Irvine Institute for Memory Impairments and Neurological Disorders (UCI MIND), Irvine, CA USA; ^4^ Institute for Memory Impairments and Neurological Disorders (UCI‐MIND), University of California, Irvine, Irvine, CA USA

## Abstract

**Background:**

Spontaneous speech is easily obtainable and has the potential to become an accessible and low‐cost marker for cognitive function. The time‐consuming and labor‐intensive nature of speech analysis has been a major obstacle to utilizing this promising tool. This study uses a novel transformer‐based methodology to explore associations between spontaneous speech language features and global cognition.

**Method:**

Speech recordings were obtained from participants with clinical diagnoses of mild cognitive impairment (MCI), dementia, and cognitively unimpaired, from the Alzheimer’s Disease Research Center (ADRC) at University of California, Irvine. Audio samples were denoised and transcribed using our transformer‐based model (Figure 1). The model conducted association analyses between global cognition, measured by Montreal Cognitive Assessment (MoCA) scores, and 5 linguistic features pre‐specified based on popularity in language analysis. Features were extracted from transcripts via Bidirectional Encoder Representations from Transformers (BERT), which assigns each word a special “token” (tokenizing), translates “tokens” into a numerical format for computer comprehension (encoding), and generates unique identifiers (embeddings) that mathematically capture meanings and relationships between words. A linear regression (LR) model was then trained on power‐transformed BERT‐extracted linguistic features and its performance was tested against both untransformed and power‐transformed data without BERT feature extraction using the same linguistic features.

**Results:**

In a cohort comprising 73 healthy controls and 12 individuals with MCI or dementia, our analysis, utilizing pre‐specified linguistic features and leveraging BERT for enhancing feature extraction, revealed that vocabulary richness (**p** = 0.009), average word length (**p** = 0.005), and semantic coherence (**p** = 0.047) were significantly associated with MoCA scores (Table 1). The non‐BERT untransformed model found no significant associations with MoCA while the non‐BERT power‐transformed model found only one significant association: average word length (**p** = 0.016).

**Conclusion:**

Our study's novel approach of employing BERT for linguistic feature extraction in association analysis increased the number of pre‐selected speech features significantly associated with global cognition, namely vocabulary richness, average word length, and semantic coherence.

This improvement in association detection highlights the potential for better deep‐learning dementia detection methods and might lead to increased utility of spontaneous speech as an easily obtainable and scalable cognitive measure.